# Emerging insights into infective endocarditis in the era of transcatheter aortic valve implantation

**DOI:** 10.3389/fcimb.2026.1822120

**Published:** 2026-07-10

**Authors:** Alexandra Dădârlat-Pop, Adela Serban, Adrian Molnar, Alexandru Oprea, Raluca Tomoaia, Stefan-Dan Moț, Iulia Dorli Demetra Moț, Horia Rosianu

**Affiliations:** 1 Cardiology Department, Heart Institute Niculae Stăncioiu, Cluj-Napoca, Romania; 2 Iuliu Haţieganu University of Medicine and Pharmacy, Cluj-Napoca, Romania; 3 Cardiovascular Surgery Department, Heart Institute Niculae Stăncioiu, Cluj-Napoca, Romania; 4 Cardiology Department, Rehabilitation Hospital, Cluj-Napoca, Romania

**Keywords:** controversial management, high surgical risk, infective endocarditis, particularities, percutaneous aortic valve implantation

## Abstract

With the rapid expansion of transcatheter aortic valve implantation (TAVI), infective endocarditis (IE) involving transcatheter prostheses has emerged as a major clinical challenge associated with high morbidity and mortality. Although the overall incidence of TAVI-associated IE remains relatively low, outcomes are poor, particularly in elderly and frail patients with multiple comorbidities. Importantly, TAVI-associated IE differs from surgical prosthetic valve endocarditis in several aspects, including patient profile, microbiological characteristics, imaging findings, and therapeutic management. This contemporary review summarizes recent evidence regarding the epidemiology, microbiology, multimodality imaging, management strategies, and prognosis of IE following TAVI. Particular emphasis is placed on the prominent role of enterococci, the limitations of conventional echocardiography, and the growing importance of multimodality imaging techniques such as cardiac CT and [¹^8^F]-FDG PET/CT for diagnosis and detection of perivalvular complications. Current evidence regarding surgical versus conservative management remains limited and strongly influenced by patient selection and operative risk, making individualized Heart Team decision-making essential. Despite advances in transcatheter therapies and imaging modalities, TAVI-associated IE continues to carry very high short- and long-term mortality. Improved preventive strategies, earlier diagnosis, optimized multimodality imaging algorithms, and better selection of patients for surgical intervention are needed to improve outcomes in this high-risk population.

## Introduction

Despite technological advances associated with the TAVI procedure, IE remains a major and extremely serious complication. Moreover, the number of TAVI-associated IE cases is expected to rise due to the increasing use of TAVI and its expansion to younger patients with longer life expectancy (Iglesias-Varea et al., 2025).

The present review aims not only to summarize recent evidence regarding infective endocarditis after TAVI, but also to emphasize the contemporary multidisciplinary challenges associated with this entity, particularly the integration of multimodality imaging, microbiological characterization, and individualized Heart Team decision-making regarding medical versus surgical management.

## 1 Why TAVI-IE is different from SAVR-PVE?

TAVI- related IE continues to represent a major challenge in terms of both diagnosis and therapeutic management. Evidence from the literature remains highly conflicting, and the choice between conservative and surgical interventions in these patients remains a subject of considerable debate. IE following TAVI continues to have a reported incidence ranging from 0.5% to 4.4% and a one-year mortality rate reaching up to 75% (Fukuhara et al., 2025).

Important trials comparing the development rate of IE after TAVI versus surgical valve replacement have shown similar incidences of IE after both types of valve replacement (Butt et al., 2019; del Val et al., 2023). Moreover, a meta-analysis of the most relevant randomized trials highlighted a slight trend toward an increased incidence of IE after TAVI in patients with intermediate surgical risk (Ando et al., 2019). It was also observed that the highest risk for IE occurs in the first 100 days after TAVI, being 6 times higher than the risk of IE 1 year after TAVI (del Val et al., 2023). Also, another multicentric study showed that IE occurred during the first year after TAVI in nine out of ten patients (Martínez-Sellés et al., 2016). However, the minimalist approach in most centers has recently led to a decrease in the rate of early IE after TAVI. Interestingly, the data from the latest trials, which included patients with low surgical risk who underwent TAVI, show a very low incidence of IE (del Val et al., 2021).

**Table 1** summarizes the most recent evidence on IE following TAVI. Relevant studies published between January 2023 and November 2025 were identified through searches of PubMed/MEDLINE, Scopus, and Google Scholar using combinations of the following keywords: “TAVI”, “TAVR”, “infective endocarditis”, “prosthetic valve endocarditis”, “transcatheter aortic valve implantation”, “multimodality imaging”, and “surgical management”. Priority was given to observational registries, multicenter cohort studies, meta-analyses, systematic reviews, and clinically relevant imaging or surgical series addressing epidemiology, microbiology, diagnosis, imaging, management, and outcomes of post-TAVI infective endocarditis. Case reports and studies lacking sufficient clinical data were excluded unless considered highly illustrative for specific imaging or management aspects.

**Table 1:** Main results from the most recent studies on IE following TAVI.

Study	Design/population	Main results	Consistent messages
Alabbadi et al., 2025 (Alabbadi et al., 2025)	US Medicare trend analysis of post-TAVI IE over 2013–2022	1-year IE incidence after TAVI fell from 20.0/1000 person-years in 2013 to 13.1/1000 in 2021. The decline occurred mainly after 2019. 30-day mortality after TAVI-IE did not improve, although explant/reintervention became more frequent over time.	IE risk after TAVI may be improving at a population level, but once IE occurs it still carries major early mortality.
Kutz et al., 2025 (Kutz et al., 2025)	Swiss nationwide cohort; adults undergoing TAVI vs bioprosthetic SAVR vs mechanical SAVR (2012–2021), propensity-matched analyses	IE incidence was highest in the first 3 months after valve intervention. After matching, TAVI had a higher IE rate than bioprosthetic SAVR (HR 1.56, 95% CI 1.12–2.18).	The early post-procedural period is the highest-risk window, and in this cohort TAVI carried more IE risk than bioSAVR.
Codner et al., 2025 (Codner et al., 2025)	Tertiary-center series of 60 post-TAVI IEreferrals (2016–2024)	7/60 (11.7%) had infected ascending aortic false aneurysm/aortitis. These lesions were identified only on cardiac CT, not echo. Most underwent surgical explant and aortic repair/replacement.	In suspected post-TAVI IE with persistent sepsis or unclear anatomy, cardiac CT is crucial, especially for detecting periaortic extension/pseudoaneurysm.
Reiter et al., 2025 (Reiter et al., 2025)	Single-center surgical case-control study of 50 patients undergoing surgery for IE after TAVI (2008–2023)	Among surgically treated patients, 30-day survival was 79.8% and 1-year survival 67.4%. Higher pre-op risk scores and postoperative dialysis were associated with perioperative death.	Surgery for post-TAVI IE is feasible but high-risk; outcomes depend strongly on baseline risk and referral to experienced centers.
Magouliotis et al., 2025 (Magouliotis et al., 2025)	Systematic review/meta-analysis of 3 studies, 1557 TAVI-IE patients comparing surgical vs medical treatment	Only 10% underwent surgery. 30-day mortality was similar between surgical and medical groups overall (9.7% vs 8.4%), and pooled 1-year survival was not statistically different; however, some single-center data suggested better survival with surgery in selected patients. Common surgical indications were severe valve dysfunction, root abscess, and large vegetations.	Current comparative evidence remains limited and affected by selection bias.
Lourtet-Hascoët et al., 2025 (Lourtet-Hascoët et al., 2025)	Prospective microbiology study of 100 TAVI patients; cutaneous cultures at puncture site	Before skin cleansing, puncture-site flora commonly included coagulase-negative staphylococci (82%) and enterococci (21%). Authors argue these data support considering amoxicillin-clavulanate prophylaxis to better cover enterococci.	Adds biologic support to the idea that enterococcal coverage matters in TAVI prophylaxis/prevention strategies.
Panagides et al., 2024 (Panagides et al., 2024)	International registry comparison of IE after TAVI vs after SAVR; matched analysis	In matched patients, vegetations were seen more often after TAVI (82% vs 62.5%), while new moderate/severe aortic regurgitation was more common after surgical bioprostheses (43.4% vs 13.5%). Authors concluded that presentation, microbiology, and treatment differ, but 1-year mortality remained high and similar between groups.	Post-TAVI IE is not identical to post-SAVR IE; imaging phenotype and management patterns differ, but prognosis remains poor in both.
Ried et al., 2024 (Ried et al., 2024)	Single-center cohort of all isolated AVR procedures (SAVR n=3447, TAVI n=2269) with subsequent PVE analysis	Reported PVE incidence did not differ significantlybetween SAVR and TAVI in this cohort. Staphylococci and enterococci were the most common pathogens. A polymer-containing TAVI prosthesis had higher PVE risk(HR 4.3). In this series, TAVI-PVE patients treated with antibiotics alone had higher 1-year survival than those undergoing additional surgery (90.9% vs 33.3%), though this is highly vulnerable to selection bias.	Findings regarding treatment strategy should be interpreted cautiously because of potential selection bias.
Strange et al., 2023 (Strange et al., 2023)	Danish nationwide registry study of 273 IE after TAVI cases	TAVI-IE patients were older and frailer; enterococci (27.1%) were prominent, and blood-culture-negative IE was uncommon (5.5%). 5-year unadjusted mortality was 75.2%. Authors suggested future prophylaxis strategies should consider enterococcal coverage.	Large contemporary registry highlighting the predominance of enterococci and the high long-term mortality associated with post-TAVI IE.

Overall, the included studies are predominantly observational in design, comprising large national registry analyses, multicenter cohorts, and single-center surgical or imaging-focused series. Sample sizes vary widely, ranging from fewer than 100 patients in detailed mechanistic or surgical studies to several thousand patients in nationwide datasets.

Across studies, the incidence of post-TAVI IE remains relatively low but clinically significant. Recent longitudinal data suggest a declining incidence over time, particularly after 2019, although this trend has not translated into meaningful improvements in short-term mortality.

Patient-related risk factors: younger age, male sex, increased BMI, diabetes mellitus, COPD, chronic kidney disease, previous infective endocarditis, pulmonary hypertension, coagulopathies, liver disease, atrial fibrillation, peripheral arterial disease, critical preoperative status, blood transfusions during hospitalization, anemia (Dădârlat-Pop et al., 2020; Iglesias-Varea et al., 2025).

Procedure-related risk factors: moderate/severe residual aortic regurgitation, low implantation of the prosthesis, vascular complications, valve-in-valve procedures, elevated post-TAVI gradients (del Val et al., 2023).

IE following TAVI is likely multifactorial. Potential contributing factors include suboptimal sterility conditions in catheterization laboratories and the high-risk clinical profile of patients undergoing the procedure, who frequently present with diabetes mellitus, immunosuppression, or chronic kidney disease (Tinica et al., 2020). Procedural aspects also play a significant role, as valve malposition requiring retrieval and reimplantation may result in endothelial injury and damage to valvular leaflets, thereby facilitating bacterial adherence. Inadequate patient education regarding post-procedural antibiotic prophylaxis and delayed or incomplete endothelialization of the bioprosthetic valve may further increase susceptibility to infection (Amat-Santos et al., 2015). Although uncommon, concomitant infections can serve as a source of bacteremia (Fortmeier and Rudolph, 2022). Additionally, residual paravalvular leaks may promote infective endocarditis by generating high-velocity jet lesions that act as a nidus during episodes of transient bacteremia. Finally, the need for endotracheal intubation and the use of self-expanding valve prostheses have been more frequently associated with the development of infective endocarditis after TAVI (Lanz et al., 2021).

### Microbiology

Microbiological patterns in post-TAVI IE differ somewhat from those observed in conventional prosthetic valve endocarditis. Although staphylococci remain among the most prevalent pathogens overall, contemporary TAVI registries have consistently highlighted the prominent role of enterococci, particularly during the early post-procedural period. This distribution may relate to transfemoral access, elderly patient populations, and healthcare-associated exposure. Nevertheless, microbiological profiles vary between cohorts, emphasizing the importance of local epidemiology and individualized antimicrobial strategies. Studies indicate that approximately one quarter of patients with post-TAVI IE are affected by enterococcal infections due to the transfemoral approach, in contrast to only about 10% of cases occurring after conventional surgical valve replacement (Khan et al., 2020; Zakhour et al., 2023). Also, other pathogenic agents, such as: *Staphylococcus aureus*, coagulase-negative staphylococci or streptococci are much less frequently involved in the development of post- TAVI IE and IE with negative blood cultures is rare (<5%) (Zakhour et al., 2023).

This difference highlights a distinct microbiological profile of IE associated with TAVI in comparison with surgical prosthetic valve IE – **Table 2**. Compared with surgical prosthetic valve endocarditis, TAVI-associated IE is characterized by a higher proportion of enterococcal infections.

**Table 2:** Comparison of microbiological profile in TAVI vs SAVR infective endocarditis.

Pathogen group	Post-TAVI IE	SAVR-PVE	Key differences / interpretation
Enterococci	~20–30% (high)	~10–20% (moderate)	More frequent in TAVI; likely related to femoral access, older population, and genitourinary sources
Staphylococcus aureus	~15–25%	~15–25%	Similar frequency, but remains major driver of early, severe IE in both
Coagulase-negative staphylococci (CoNS)	~15–25%	~15–25%	Important in both due to prosthetic material and biofilm formation
Staphylococci (overall)	~30–45%	~30–50% (dominant group)	More dominant in SAVR, especially early postoperative IE
Streptococci	~10–20%	~15–30%	More common in SAVR, reflecting community-acquired late IE
Gram-negative (non-HACEK)	<10%	<10%	Uncommon in both; typically healthcare-associated
HACEK organisms	Rare (<5%)	Rare (<5%)	Similar, low prevalence
Fungi	Rare (<5%)	Rare (<5%)	Rare but very high mortality in both
Culture-negative IE	~5–10%	~5–15% (higher)	More frequent in SAVR, possibly due to perioperative antibiotics and diagnostic challenges

Ranges shown in **Table 2** represent approximate values derived from contemporary observational registries and cohort studies included in this review and should not be interpreted as pooled meta-analytic estimates.

### Clinical presentation

Fever is present in approximately 80% of cases; however, about 40% of patients with post-TAVI IE may develop new heart failure symptoms (Zakhour et al., 2023). Thirteen percent have manifestations due to systemic embolization, but unfortunately some of these patients may present with atypical or nonspecific symptoms, which can delay the diagnosis. Moreover, the modified Duke criteria demonstrate a lower diagnostic accuracy in IE following TAVI compared with native valve IE (Delgado et al., 2023). This limitation is mainly attributable to the reduced sensitivity of echocardiography in the presence of transcatheter valve prostheses, including acoustic shadowing from the prosthetic stent frame and difficulty detecting vegetations or perivalvular complications. In contrast, culture-negative IE appears relatively uncommon in contemporary TAVI-IE cohorts and should not be presented as a predominant diagnostic limitation (Delgado et al., 2023).

## 2 Why echocardiography alone is insufficient?

The 2023 European Society of Cardiology (ESC) diagnostic criteria for infective endocarditis have expanded the role of multimodality imaging, incorporating echocardiography, cardiac CT, [¹^8^F]-FDG PET/CT, and white blood cell (WBC) SPECT/CT as major diagnostic criteria in cases of prosthetic valve infection (Vinod et al., 2025). These recommendations are particularly relevant in TAVI-associated infective endocarditis, where conventional echocardiography alone may have limited sensitivity because of metallic stent-related acoustic shadowing and difficulty visualizing vegetations or perivalvular complications. However, important limitations remain. Early after TAVI implantaton, inflammatory post-procedural FDG uptake may reduce the specificity of PET/CT imaging, and differentiation between infection and postoperative inflammatory changes may be challenging. Therefore, despite the important advances incorporated into the 2023 ESC criteria, diagnosis of TAVI-associated infective endocarditis still requires careful integration of clinical, microbiological, and multimodality imaging findings within a multidisciplinary Heart Team approach. So, transthoracic (TTE) and transesophageal echocardiography (TEE) remain the primary imaging modalities for the assessment of IE following TAVI, and are widely available in most centers. In clinical practice, both TTE and TEE are usually performed to optimize diagnostic accuracy. Additionally, ^18F-fluorodeoxyglucose positron emission tomography/computed tomography (^18F-FDG PET/CT) is particularly valuable in the diagnostic work-up of prosthetic valve endocarditis, including graft infections, especially when TEE findings are inconclusive (Miranda et al., 2018).

**Figure 1:**
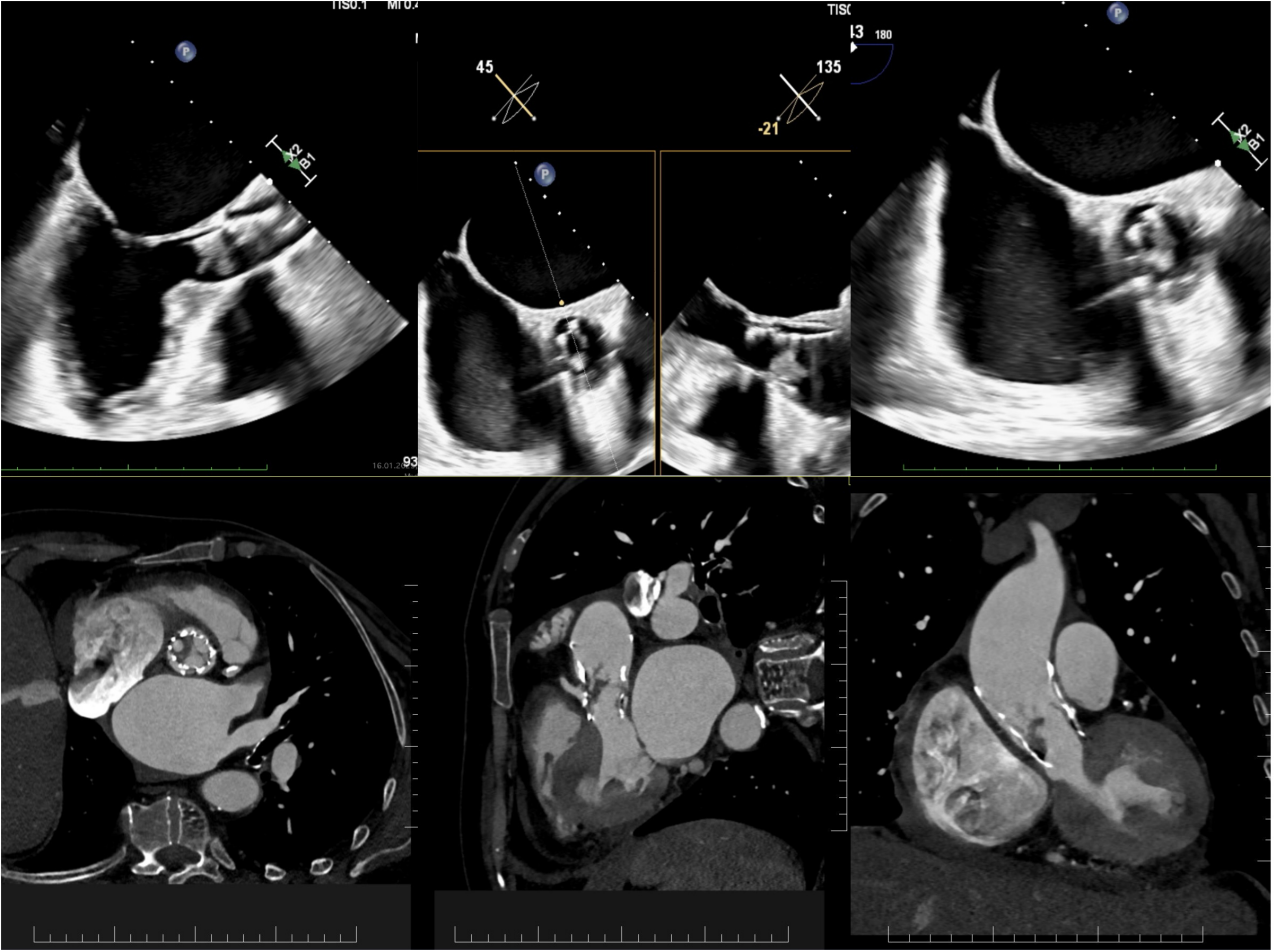
Infective endocarditis occurring three months after implantation of a 27-mm Navitor prosthesis caused by *Enterococcus faecalis*. TEE and cardiac CT demonstrated large vegetations (red arrow) and leaflet thickening (white arrows), however, the prosthetic valve was not dysfunctional, and no periprosthetic complications were identified – **Supplementary Movie 1**.

Multimodality imaging plays a central role in the diagnosis and evaluation of TAVI-associated infective endocarditis, particularly when echocardiographic findings are inconclusive. Cardiac CT is especially useful for detecting perivalvular complications, including abscesses, pseudoaneurysms, fistulas, leaflet thickening, and prosthetic dehiscence, and may identify lesions not visible on echocardiography, as you can see in **Figure 1**. [¹^8^F]-FDG PET/CT is most valuable in cases of suspected prosthetic valve infection with nondiagnostic echocardiography, as it can detect abnormal metabolic activity around the prosthesis and extracardiac septic embolic foci. However, its specificity may be reduced during the early post-TAVI period because of inflammatory postoperative FDG uptake. White blood cell (WBC) SPECT/CT may provide additional diagnostic value when PET/CT findings are equivocal or when differentiation between infection and sterile inflammation is challenging. Brain MRI is particularly sensitive for detecting silent cerebral embolic lesions and neurological complications, which may influence both prognosis and therapeutic decision-making. Whole-body CT is useful for identifying extracardiac septic embolization, including splenic, renal, and peripheral embolic complications, as well as occult infectious foci. Therefore, imaging selection should be individualized according to clinical presentation, timing after TAVI implantation, microbiological findings, and suspected complications.

Examples of multimodality imaging in post-TAVI IE:

### Imaging of systemic complications

Cross-sectional imaging, including whole-body CT or magnetic resonance imaging (MRI), plays a complementary role in detecting systemic complications of post-TAVI IE, such as cerebral embolic events, splenic or renal infarctions, and mycotic aneurysms. Brain MRI is particularly sensitive for silent embolic lesions and may influence therapeutic decisions.

The diagnostic accuracy of TEE is lower in TAVI prostheses compared with surgically implanted valves, and conventional echocardiographic parameters may not always be applicable in this patient population (San et al., 2019). Studies have shown that vegetations could be visualized by TEE in only approximately 25% of patients (Miranda et al., 2018; San et al., 2019). Therefore, in patients with a high clinical suspicion, a negative TEE does not exclude infective endocarditis following TAVI.

The use of PET/CT in the immediate postoperative period after TAVI for patients with suspected IE is controversial due to the associated inflammatory response (Wahadat et al., 2021). FDG uptake immediately following TAVI is typically uniform and circumferential, reflecting post-procedural inflammation, and generally resolves within approximately one month. In contrast, patients with post-TAVI infective endocarditis exhibit focal or multifocal FDG uptake patterns, distinguishing infection from normal post-procedural changes (San et al., 2019; Panagides et al., 2022).

### Particularities of post-TAVI IE

**Figure 2:**
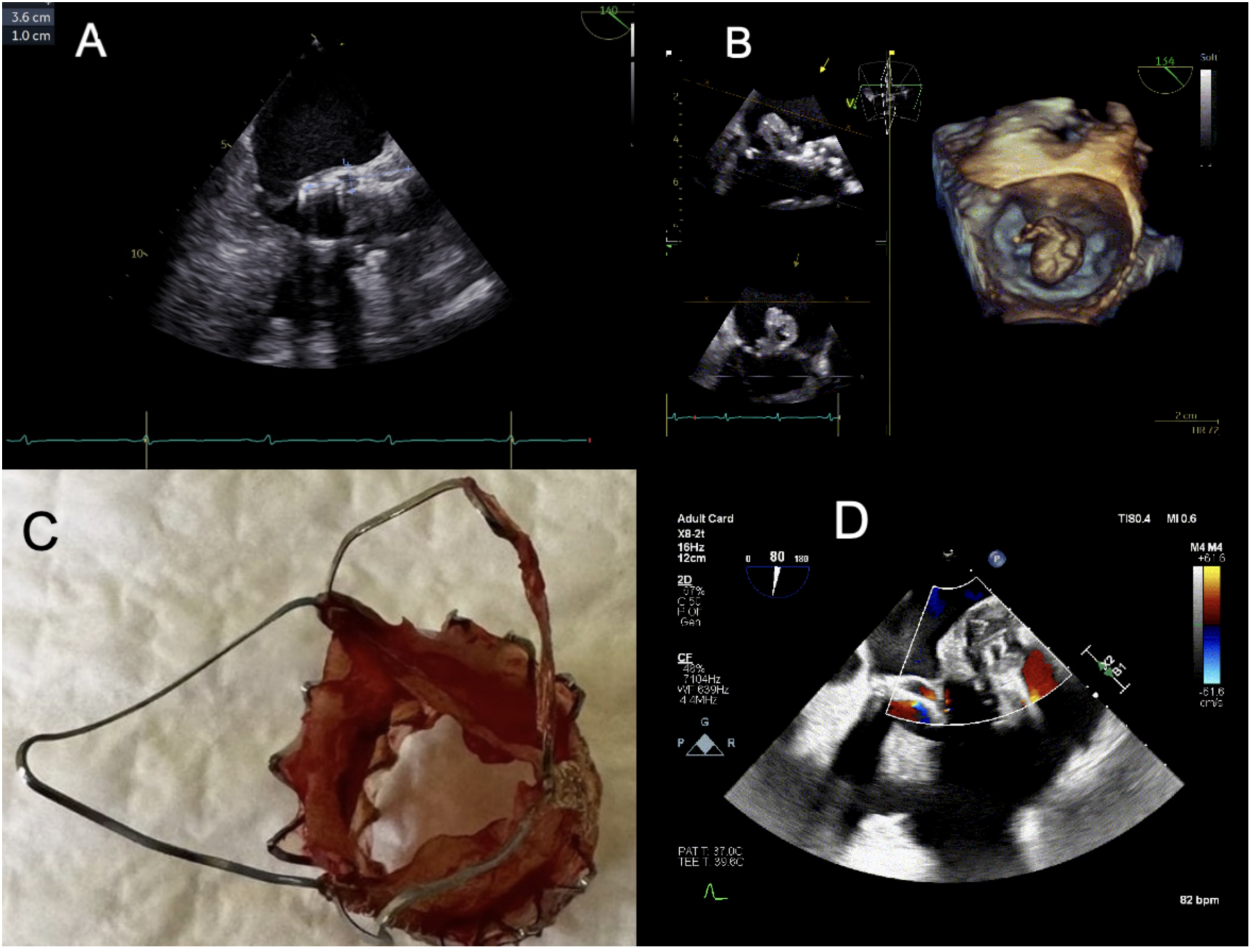
IE of the Accurate Neo 2 TAVI prosthesis **(A)** and mitral valve **(B)** occurring nine months after implantation caused by *Enterococcus faecalis*. TEE revealed a giant, heterogeneous, on the atrial side of the anterior mitral leaflet [**(b)** and **Supplementary Movie 2**], accompanied by moderate mitral regurgitation. Additionally, a paravalvular TAVI abscess is described **(A)**, involving the mitro-aortic continuity and fistulizing into the left ventricular outflow tract and the left atrium –**Supplementary Movie 3**. ECG-newly developed complete atrioventricular block. **(C)** - Intraoperative appearance of the explanted TAVI prosthesis. Mitral and aortic valve replacement were performed, along with reconstruction of the ascending aortic wall using a bovine pericardial patch. The immediate postoperative course was favorable. TEE at the 3-month follow-up showing a heterogeneous, echogenic mass localized periprosthetically on the posterior wall of the ascending aorta. No color Doppler signal was detected – **(D)**. The mass collapses during systole and does not interfere with the function of the biological aortic and mitral prostheses – **Supplementary Movie 4**.

A distinctive feature of post-TAVI IE is that most vegetations are attached to the stent cells of the transcatheter valve, particularly when a self-expanding transcatheter heart valve (THV) is used (del Val et al., 2023). In approximately one-third of cases, vegetations are located outside the valve, and in some instances, vegetations may also involve other cardiac valves, such as the mitral valve (MV) – **Figure 2A**. MV-IE was associated with self-expanding THV implantation and the presence of at least moderate aortic regurgitation at discharge. Surgical treatment is quite uncommon in these patients, and they have a poor prognosis (Hadji-Turdeghal et al., 2023).

### Valve-in-valve TAVI–related IE

While earlier studies reported that prosthetic valve IE accounted for up to 5% of all IE cases, more recent investigations suggest a rising contribution, with prosthetic valve IE now representing approximately 14–20% of total IE cases (Fukuhara et al., 2025). Data regarding the rate of IE following valve-in-valve procedures are limited, and the incidence of endocarditis in these cases is unknown. However, there is evidence that this type of intervention associates a significantly higher risk of IE compared with conventional TAVI (de la Cuesta et al., 2025). This is most likely related to the greater procedural complexity, the presence of residual aortic leaks, higher residual transvalvular aortic gradient, or low implantation of the transcatheter prosthetic valve -**Figure 2**.

## 3 Why surgery remains controversial?

Post-TAVI IE is associated with markedly increased mortality, reaching up to 64% in-hospital and up to 75% at one year. The rate of associated complications is also high, underscoring the severity of this condition (de la Cuesta et al., 2025). Management of IE in both TAVI and SAVR requires a multidisciplinary endocarditis team approach, but important differences exist, as you can see in **Table 3**.

**Table 3:** Comparison of TAVI-IE versus SAVR-PVE from a heart team perspective.

Feature	TAVI-IE	SAVR-PVE
Typical patient	Older/frailer	Younger
Common pathogens	Enterococci	Staphylococci
Echo limitations	More frequent	Less severe
CT utility	Very high	Moderate
Surgery feasibility	Often limited	More feasible
Mortality	Very high	High

Surgical intervention is a cornerstone of treatment in SAVR-PVE, particularly in cases of heart failure, uncontrolled infection, or perivalvular complications, and is supported by established guideline recommendations (Delgado et al., 2023).

In contrast, surgical explantation after TAVI is less frequently performed, primarily due to the high operative risk and frailty of the patient population. As a result, many TAVI-IE patients are managed conservatively with antibiotics alone. Current evidence comparing surgical and medical management in TAVI-IE is limited and subject to significant selection bias, with no clear survival advantage consistently demonstrated for either strategy at the population level.

Guidelines recommend prompt surgical intervention in complicated cases of infective endocarditis, such as patients with heart failure, perivalvular complications, or a high risk of embolization (Mangner et al., 2022). However, these recommendations are often not applicable to post-TAVI patients due to the elevated surgical risk, contraindications to surgery, and technical challenges associated with extracting the transcatheter valve system, particularly the stent adherent to the aorta.

Currently, there is insufficient data in the literature to definitively conclude whether surgical intervention is superior to medical therapy in post-TAVI IE. For example, there are registries where most patients diagnosed with TAVI-IE were managed conservatively with antibiotic therapy alone (Takei et al., 2023). Moreover, surgical intervention did not confer a significant reduction in all-cause mortality during hospitalization or at one year. Elevated mortality rates in this cohort were predominantly influenced by patient-specific factors, the infecting pathogen, and complications arising from IE (Iglesias-Varea et al., 2025). Conversely, other reports suggest that surgical intervention may reduce mortality in selected cases (Takei et al., 2023). Therefore, the decision to pursue surgery in these patients must be individualized, taking into account the clinical status, surgical risk, and associated comorbidities.

For patients with post-TAVI IE who are managed conservatively and considered cured based on multimodal imaging, but who exhibit residual valvular dysfunction, repeat transcatheter valve-in-valve therapy may be considered within 1–3 months (Kefer et al., 2024; Borger et al., 2025). Unfortunately, there are currently no clear guideline recommendations regarding the management of patients with TAVI-associated IE. Moreover, data on IE in patients undergoing transcatheter aortic valve–in–surgical aortic valve procedures are extremely limited. Consequently, management decisions are left to the Heart Team, a process that is particularly challenging given the frailty of these patients and their high surgical risk - as summarised in **Table 3**. In most cases, antibiotic therapy guided by antimicrobial susceptibility remains the only feasible treatment option, albeit with an unfortunately high associated mortality. As discussed, approximately two-thirds of patients experience complications, and both in-hospital and 30-day mortality rates remain high (del Val et al., 2023).

### Prognosis

Despite differences in patient characteristics and management approaches, outcomes remain poor in both groups (del Val et al., 2023). Short-term mortality is high, particularly in cases involving *Staphylococcus aureus* or complicated infection. Long-term prognosis is also unfavorable, with substantial mortality reported at 1 and 5 years.

Importantly, some data suggest that although TAVI patients are older and higher risk, adjusted mortality may be comparable between TAVI-IE and SAVR-PVE, highlighting the overall severity of prosthetic valve IE regardless of intervention type (Delgado et al., 2023; del Val et al., 2023).

## 4 Why prevention matters more than ever?

Understanding the differences between TAVI-IE and SAVR associated IE has important implications for clinical practice. The prominent role of enterococci in TAVI-associated infective endocarditis suggests that current prophylactic and empiric antibiotic strategies may warrant further investigation. However, the available evidence remains largely observational and hypothesis-generating, and there is currently insufficient guideline-level evidence to support routine modification of prophylactic regimens in clinical practice. Preventive measures are therefore of paramount importance (Borger et al., 2025). Traffic in catheterization laboratories should be minimized, doors should remain closed throughout the procedure or in hybrid operating rooms, and exposure of the transcatheter system to ambient air should be reduced as much as possible. Adequate patient preparation is essential both before and after the intervention (del Val et al., 2023).

## Limitations

This review has several limitations that should be acknowledged. First, the available evidence on IE following TAVI is largely derived from observational studies, registries, and retrospective analyses, which are inherently subject to selection bias, unmeasured confounding, and heterogeneity in patient populations. Direct comparisons between TAVI-associated IE and SAVR- related IE are particularly challenging, as patients undergoing TAVI are typically older and have a higher burden of comorbidities. Second, there is substantial variability in definitions, diagnostic criteria, and reporting of outcomes across studies, including differences in the application of modified Duke criteria and the use of multimodality imaging. This heterogeneity limits the ability to perform robust quantitative comparisons and may affect the generalizability of findings.

Moreover, microbiological data are not uniformly reported, and some studies lack detailed pathogen-specific outcomes. In addition, the reported proportions of microorganisms may vary depending on geographic region, institutional practices, and prophylactic strategies. Also, evidence regarding optimal management strategies, particularly the role of surgery in TAVI-associated IE, remains limited. Comparative studies between surgical and medical treatment are scarce and often affected by significant treatment selection bias, as patients referred for surgery are typically younger or have different risk profiles compared with those managed conservatively. Because this review was designed as a narrative contemporary overview rather than a systematic review, study selection was not performed according to predefined PRISMA criteria.

Finally, as TAVI technology and indications continue to evolve, including its expansion into younger and lower-risk populations, the epidemiology and outcomes of TAVI-associated IE may change over time. Therefore, some findings from earlier studies may not fully reflect contemporary practice.

## Conclusion

TAVI-associated infective endocarditis represents a complex and increasingly relevant clinical entity characterized by a distinct microbiological profile, major diagnostic challenges, and persistently poor outcomes. Compared with SAVR-associated prosthetic valve endocarditis, TAVI-IE is more frequently associated with enterococcal infections, greater reliance on multimodality imaging, and lower rates of surgical intervention, largely reflecting the advanced age and frailty of this population. Despite important advances in transcatheter therapies and imaging modalities, mortality remains high, emphasizing the need for improved preventive strategies, earlier diagnosis, optimized multimodality imaging algorithms, and individualized multidisciplinary Heart Team management. Further prospective studies are required to better define optimal prophylactic strategies, imaging pathways, and patient selection for surgical intervention.
