# Supplementary figures and images for: Emerging insights into infective endocarditis in the era of transcatheter aortic valve implantation
Source: Front Cell Infect Microbiol. 2026 Jul 10;16:1822120. doi: 10.3389/fcimb.2026.1822120 (PMC13397106; doi:10.3389/fcimb.2026.1822120)

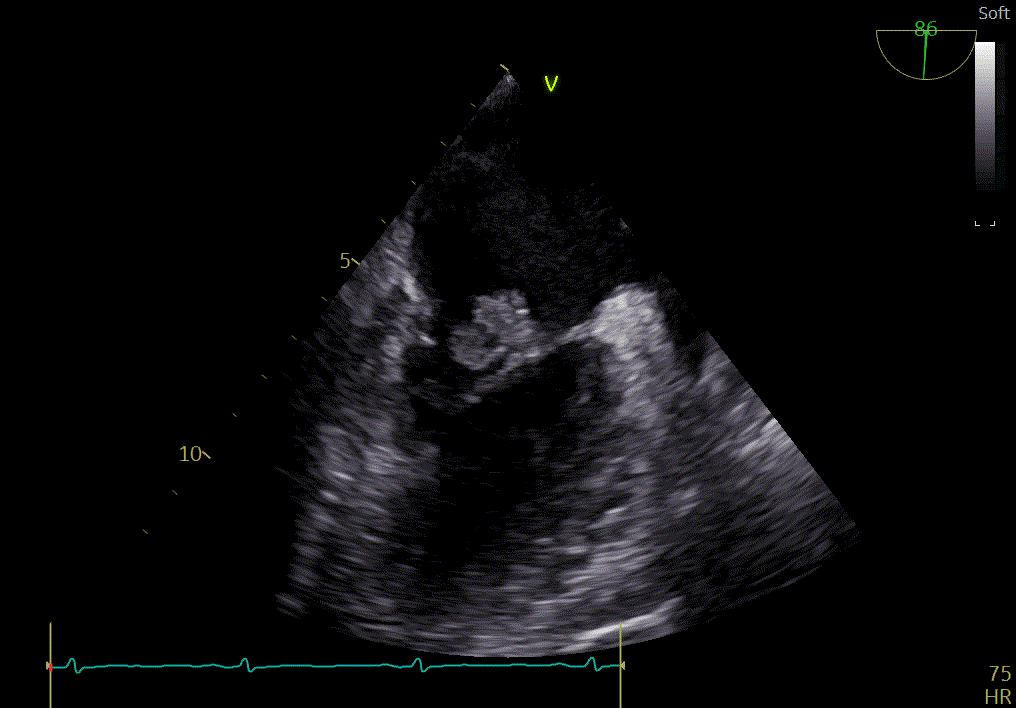

Supplement: Supplementary file 2 [file SupplementaryFile2.gif]

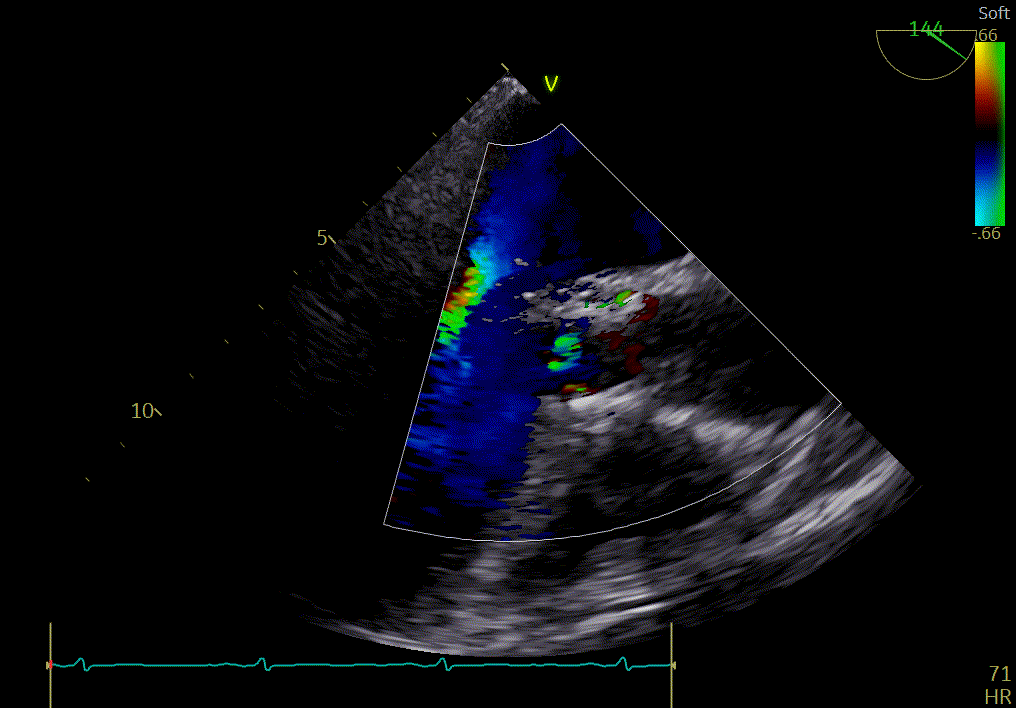

Supplement: Supplementary file 3 [file SupplementaryFile3.gif]

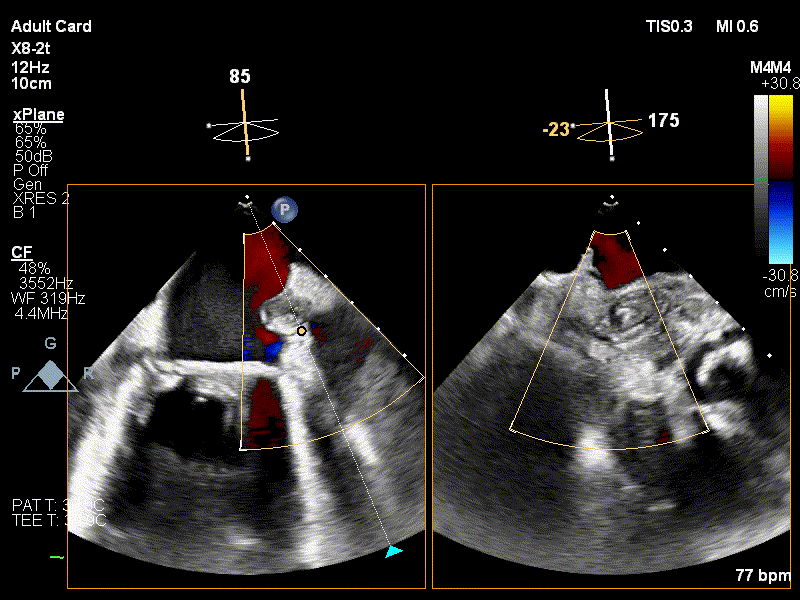

Supplement: Supplementary file 4 [file SupplementaryFile4.gif]
